# Knowledge, attitudes and skills of dementia care in general practice: a cross-sectional study in primary health settings in Beijing, China

**DOI:** 10.1186/s12875-020-01164-3

**Published:** 2020-05-16

**Authors:** Meirong Wang, Xiaojingyuan Xu, Yafang Huang, Shuang Shao, Xiaolei Chen, Jing Li, Juan Du

**Affiliations:** 1 School of General Practice and Continuing Education, Capital Medical University, No. 10, You An Men Wai Xi Tou Tiao, Beijing, 100069 China; 2 Dongfeng Community Health Service Center, Chaoyang district, Nan Shi Li Ju, Beijing, 100016 China

**Keywords:** Dementia, General practitioners, Attitudes, Knowledge

## Abstract

**Background:**

General practitioners (GPs) play a significant role in dementia care. However, the knowledge and attitudes of them towards dementia care are poorly characterized. The present study aimed to investigate GPs’ knowledge, attitudes and skills of dementia care in primary health settings in Beijing.

**Methods:**

A cross-sectional survey was conducted in 27 community health service centers (CHSCs) in Beijing. The GPs’ knowledge, attitudes and skills were assessed utilizing the Alzheimer’s Disease Knowledge Scale (ADKS), Dementia Care Attitude Scale (DCAS) and self-designed questionnaire, respectively.

**Results:**

A total of 341 participants returned the questionnaire. The overall mean score of GPs’ dementia knowledge measured by the ADKS was 21.42 (SD = 2.73) out of 30 (71.4%), GPs’ attitudes to dementia care was 36.25 (SD = 5.12) out of 50 (72.5%), and GPs’ self-confidence on dementia care skills was 53.93 (SD = 9.57) out of 75 (71.9%). GPs’ overall knowledge towards dementia care was limited and the attitudes were generally positive. They had low level recognition of their roles towards dementia care. The majority of GPs believed that dementia care was within a specialist’s domain not that of general practice.

**Conclusion:**

GPs demonstrate low levels of dementia knowledge and skills, but express generally positive attitudes towards dementia in this study. It is much needed to translate detailed dementia care handbook, and adequate dementia knowledge training for GPs into practice to improve care outcomes for people with dementia in China. In addition, dementia management should be covered in the national basic package of public health services in primary care.

## Background

Dementia is a collective name for progressive brain syndromes which affect memory, thinking, behavior and emotion. As a disease with a progressive process, dementia not only increases the disability and dependency of the elderly themselves, it also affects their caregivers’ lives. There were around 50 million people worldwide are living with dementia in 2018 and this number may increase to 152 million by 2050 [1]. China has the largest dementia population around the world. The prevalence of dementia was 5.60% (95% CI 3.50–7.60) in 2019 among the elderly aged 65 years and above [2]. The number of dementia patients will increase to 35.98 million in China by 2050 [3].

Various dementia care models in primary care in some western counties have been developed, which can be summarized as “collaborative care” or “patient-centered care” to address patients’ and caregivers’ complex healthcare needs and to enable multidisciplinary care [4]. Dementia care in primary care setting is beneficial to both dementia patients and their caregivers [5, 6]. To improve the practice, there are guidelines published in Canada, the USA and Australia for the diagnosis and management of dementia in the primary care [7–9]. For GPs, they have positive attitudes towards the diagnosis and treatment for dementia patients [10–12]. However, the barriers were still existed. For example, they were not confident in their skills on making the early diagnosis or screening patients to be referred to the specialists [11, 13, 14]. In order to address these problems, further education of GPs has been widely advocated [15].

In China, almost all dementia cases are diagnosed by neurologists in tertiary hospitals. However, they don’t provide long-term management. The referral system between hospitals and CHSCs has not been well established, which prevents dementia patients from getting dementia-specific healthcare [16]. Dementia management has not been covered in the national basic package of public health services in primary care [17]. The availability of community-based dementia-specific services is still very limited, since GPs in the CHSCs put most effort into developing medical services on the contents covered in the package [18]. Based on the conditions above and limited knowledge on dementia care, GPs could only provide basic services like simple guides for care despite they wanted to be more helpful [19, 20]. Day care and telephone services from social care organizations would provide care and emotional support to dementia patients and their families, but the quality of these services was still low since lacking supports from medical systems [21]. The World Health Organization (WHO) states that it would be challenging to intervene without the effective involvement of primary care [22]. According to World Alzheimer report 2016, primary care workforce can alleviate resource, especially critical for low- and middle-income countries [23]. Providing care for dementia patients in primary care is a predictable trend in China.

Beijing is the capital city of China with over 3.33 million people. About 24.5% of them are aged 60 years or above [24]. Approximately 150 thousand patients were suffered from dementia until 2015 in Beijing [25]. The number of cases is even larger now [26]. Knowing the GPs’ view of dementia and exploring whether they are competent for dementia management are basic steps to identify the underlying problems in primary care workers in Beijing, a city with large dementia population and community-based dementia care services are much needed. The objective of this study was to investigate GPs’ knowledge, attitudes and skills of dementia care in primary health settings in Beijing and to provide some references for dementia care in the future.

## Methods

### Sample and settings

Beijing totally includes six urban districts and ten rural districts. There were approximately 2.16 million elderly in urban districts, accounting for 64.9% of the elderly population in Beijing [24]. We carried out a cross-sectional questionnaire survey in all the six urban districts from July to December 2018. A two-step sampling approach was used to minimize selection bias and ensure generalizability. First, 20% of the CHSCs in each urban district were selected by simple random sampling method. Second, all the qualified GPs (specialists who finished job-transfer training program and medical college graduates who finished standardized residency training of general practice) worked in current CHSCs for at least 1 year were invited to participate in the study. A total of 27 CHSCs were selected from 132 CHSCs (see Table 1 for additional characteristics), and 350 GPs were recruited to survey.

**Table 1:** Selected CHSCs from six urban districts in Beijing

Districts	CHSCs (n)	20% of the total CHSCs (n)
Chaoyang	46	9
Dongcheng	7	2
Fengtai	22	5
Haidian	32	6
Shijingshan	10	2
Xicheng	15	3
Total	132	27

Available from: http://www.bjchs.org.cn/

### Survey instruments

There were three steps in questionnaire development. First, question lists were developed by collecting and extracting information from references. Second, several experts in related area were invited to review the questionnaire and a small sample preliminary investigation was conducted. Third, the questionnaire was adjusted according to the opinions of experts and preliminary findings before formal survey.

The final questionnaire included five parts. The first part was to collect GPs’ demographic information on gender, age, educational degree, work experience and professional title. The second part was to collect GPs’ working, training and caring experience on dementia, such as number of dementia patients and caregivers they treated, caring experience on dementia patients, available drugs for dementia in the CHSC they working at, dementia related training experience, etc.

The third part of the questionnaire was to assess GPs’ dementia knowledge with the ADKS for its ease of use, demonstrated reliability and validity [27]. The 30 true/false items in the ADKS were conceptually divided into seven domains: life impact (3 items), assessment and diagnosis (4 items), symptoms (4 items), disease progression (4 items), treatment and management (4 items), caregiving (5 items), risk factors (6 items). The score was calculated by summing the correct items together, ranging from 0 to 30. A higher total score indicated better knowledge. Mean correct rate indicated the average of correct rate of each item in the domain. The Chinese version of the ADKS was strictly translated using the revised version of Brislin Translation Model. It had an acceptable psychometric property and could be used to measure the related knowledge among patients, caregivers and medical staffs in Chinese population [28].

The fourth part of the questionnaire was to collect GPs’ attitudes on the management of demented patients using the DCAS [29]. It included 10 items graded on a 5-point Likert-type scale with responses varying from “strongly disagree” to “strongly agree”. When calculating the total score, 1 indicated the most negative attitude while 5 indicated the most positive. Four of ten items were negatively worded and were reversed in a definite order. The total score was ranged from 10 to 50. Higher score indicated more positive attitudes. The Chinese version of DCAS was used in this study [30].

The fifth part of the questionnaire was to assess GPs’ confidence on their dementia care skills using a self-designed questionnaire. The 15 items were measured via a 5-point Likert-type scale (1 = I can’t do it at all, 2 = I can’t do it well, 3 = I’m not sure, 4 = I can do it probably, 5 = I can do it very well). The total score ranges from 15 to 75. Higher score indicated that the GP is more confident in his/her dementia care skills. The Cronbach’s α of the all 15 items was 0.947, indicating good internal consistency. Principle component analysis was conducted to confirmed the factorability of the data set (KMO = 0.930, Bartlett’s test of Sphericity *P* < 0.001). The factor analysis revealed 2 factors. One was comprised by the 7 items representing pre-diagnosis skills, and the other was comprised by the 8 items on post-diagnosis skills. The cumulative contribution rate was 70.682%.

### Data collection and quality control

The participants filled the questionnaires through internet. The link of the questionnaire was first distributed to the managers of the 27 CHSCs. Study managers shared the link to all the GPs in the CHSCs who meet all the criteria above. To ensure the participants completed the questionnaires seriously, a minimum time limit was set up. Therefore, the participant was not able to submit the questionnaire if he/she only spend too short time. The questionnaire cannot be submitted unless all the questions were answered.

### Statistical analysis

Data of questionnaires were imported into Excel 2016, and was checked by two researchers. The sample and variables were described by descriptive statistics including mean with standard deviations (SD) and median with inter-quartile range (IQR). The Kolmogorov-Smirnov test was used to assess normality of distribution of the knowledge, attitudes, and management scores in different demographic characteristics. Spearman correlation analyses were used to measure the correlation between the scores of knowledge, attitudes, and skills. All analyses were conducted using the IBM Statistical Package for Social Science software program (IBM-SPSS) Version 20.0 for Windows and all the tests are two sided, with statistical significance set at 0.05.

## Results

Of the 350 questionnaires administered, 341 questionnaires were returned (response rate 97.4%); 9 declined with no excuse. The GPs aged between 27 and 56 years (mean = 39 years, SD = 6.7) and their work experience was between 1 and 35 years (mean = 16 years, SD = 7.8). Detailed demographic information is showed in Table 2.

**Table 2:** Demographic data of GPs (*n* = 341)

Characteristics	n (%)	Characteristics	n (%)
GPs in each district		Education	
Chaoyang	117 (34.3)	Master degree or above	52 (15.2)
Dongcheng	15 (4.4)	Bachelor	258 (75.7)
Fengtai	90 (26.4)	College degree or below	31 (9.1)
Haidian	48 (14.1)	Work experience	
Shijingshan	11 (3.2)	Under 10 years	83 (24.3)
Xicheng	60 (17.6)	10–19 years	139 (40.8)
Gender		20–29 years	104 (30.5)
Male	72 (21.1)	30 years and above	15 (4.4)
Female	269 (78.9)	Professional title	
Age		Primary title	64 (18.8)
Under 30 years	30 (8.8)	Intermediate title	189 (55.4)
30–39 years	152 (44.6)	Senior title	88 (25.8)
40–49 years	139 (40.7)		
50 years and above	20 (5.9)		

### The supply status of anti-dementia drugs in CHSCs where the recruited GPs worked

About 105 (30.8%) of the GPs expressed that the CHSCs where they worked could provide donepezil, and 28 (8.2%) of them indicated they could provide memantine. Whereas 217 (63.7%) of GPs were not sure if they had anti-dementia drugs in their work places (see Table 3 for details).

**Table 3:** The supply status of anti-dementia drugs in CHSCs (*n* = 341)

Items	Number of GPs who could provide anti-dementia drugs (%)
Donepezil	105 (30.8)
Cappastin	5 (1.5)
Galantamine	3 (0.9)
Memantine	28 (8.2)
Others	26 (7.6)

### GPs’ work experience on dementia

The median number of dementia patients, caregivers of dementia patients and patients with possibility of dementia a GP had met in the past 3 months were 2 (IQR: 0–3), 3 (IQR: 1–5) and 2 (IQR: 0–5), respectively. And 59 (17.3%) GPs hadn’t met any of them in the past 3 months. Other details are showed in Table 4. There were 39 GPs (11.4%) had a dementia family member and 21 (53.8%) out of them had participated in caring for the dementia family member.

**Table 4:** GPs’ work experience on dementia (*n* = 341)

People you had met in the past 3 months	n (%)
Diagnosed dementia patients	
Never met	127 (37.2)
1–5	165 (48.4)
6–10	29 (8.5)
11 and above	20 (5.9)
Caregivers of diagnosed dementia patient	
Never met	77 (22.6)
1–5	171 (50.1)
6–10	40 (11.8)
11 and above	53 (15.5)
Patients may have dementia	
Never met	95 (27.9)
1–5	173 (50.7)
6–10	35 (10.3)
11 and above	38 (11.1)

Only 52 (15.2%) GPs received dementia related training in the past 1 year. Whereas, 189 (55.4%) of them got knowledge from professional books, 155 (45.5%) got it from professional websites, 138 (40.5%) got it from literatures and guidelines, 141 (41.3%) got it from academic conferences, 116 (34.0%) got it from TV programs, 87 (25.5%) got it from posters and brochures, and 76 (22.3%) got it from colleagues and specialists.

### GPs’ dementia knowledge

The overall mean score of GPs’ dementia knowledge measured by the ADKS was 21.42 (SD = 2.73) out of 30 (71.4%). Items related to “treatment and management” were answered best, the mean correct rate was 85.2%, while those related to “caregiving” were the poorest, the mean correct rate was 55.1%. The items with the best correct rate were “A person with Alzheimer’s disease becomes increasingly likely to fall down as the disease gets worse”, “Alzheimer’s disease is one type of dementia” and “Genes can only partially account for the development of Alzheimer’s disease”, while the poorest responses were “If trouble with memory and confused thinking appears suddenly, it is likely due to Alzheimer’s disease”, “It has been scientifically proven that mental exercise can prevent a person from getting Alzheimer’s disease” and “Tremor or shaking of the hands or arms is a common symptom in people with Alzheimer’s disease”. More details are showed in Table 5 and Table 6.

**Table 5:** GPs’ dementia knowledge measured by the ADKS (by domains)

Domains	Items	Range of total score	Mean ± SD	Mean correct rate (%)
Treatment and management	9,12,24,29	0–4	3.41 ± 0.71	85.2
Life impact	1,11,28	0–3	2.43 ± 0.68	80.8
Course	3,8,14,17	0–4	3.11 ± 0.69	77.9
Assessment	4,10,20,21	0–4	2.93 ± 0.60	73.4
Risk factor	2,13,18,25,26,27	0–6	4.28 ± 1.03	71.3
Symptom	19,22,23,30	0–4	2.50 ± 0.88	62.5
Caregiving	5,6,7,15,16	0–5	2.75 ± 1.12	55.1

**Table 6:** GPs’ dementia knowledge measured by the ADKS (by items, *n* = 341)

Items	Number of GPs answered correctly (%)
1. People with Alzheimer’s disease are particularly prone to depression.	312 (91.5)
2. It has been scientifically proven that mental exercise can prevent a person from getting Alzheimer’s disease.	76 (22.3)
3. After symptoms of Alzheimer’s disease appear, the average life expectancy is 6 to 12 years.	288 (84.5)
4. When a person with Alzheimer’s disease becomes agitated, a medical examination might reveal other health problems that caused the agitation.	307 (90.0)
5. People with Alzheimer’s disease do best with simple, instructions given one step at a time.	255 (74.8)
6. When people with Alzheimer’s disease begin to have difficulty taking care of themselves, caregivers should take over right away.	127 (37.2)
7. If a person with Alzheimer’s disease becomes alert and agitated at night, a good strategy is to try to make sure that the person gets plenty of physical activity during the day.	254 (74.5)
8. In rare cases, people have recovered from Alzheimer’s disease.	132 (38.7)
9. People whose Alzheimer’s disease is not yet severe can benefit from psychotherapy for depression and anxiety.	319 (93.5)
10. If trouble with memory and confused thinking appears suddenly, it is likely due to Alzheimer’s disease.	63 (18.5)
11. Most people with Alzheimer’s disease live in nursing homes.	220 (64.5)
12. Poor nutrition can make the symptoms of Alzheimer’s disease worse.	321 (94.1)
13. People in their 30s can have Alzheimer’s disease.	255 (74.8)
14. A person with Alzheimer’s disease becomes increasingly likely to fall down as the disease gets worse.	329 (96.5)
15. When people with Alzheimer’s disease repeat the same question or story several times, it is helpful to remind them that they are repeating themselves.	109 (32.0)
16. Once people have Alzheimer’s disease, they are no longer capable of making informed decisions about their own care.	194 (56.9)
17. Eventually, a person with Alzheimer’s disease will need 24-h supervision.	313 (91.8)
18. Having high cholesterol may increase a person’s risk of developing Alzheimer’s disease.	300 (88.0)
19. Tremor or shaking of the hands or arms is a common symptom in people with Alzheimer’s disease.	100 (29.3)
20. Symptoms of severe depression can be mistaken for symptoms of Alzheimer’s disease.	302 (88.6)
21. Alzheimer’s disease is one type of dementia.	329 (96.5)
22. Trouble handling money or paying bills is a common early symptom of Alzheimer’s disease.	285 (83.6)
23. One symptom that can occur with Alzheimer’s disease is believing that other people are stealing one’s things.	242 (71.0)
24. When a person has Alzheimer’s disease, using reminder notes is a crutch that can contribute to decline.	236 (69.2)
25. Prescription drugs that prevent Alzheimer’s disease are available.	198 (58.1)
26. Having high blood pressure may increase a person’s risk of developing Alzheimer’s disease.	301 (88.3)
27. Genes can only partially account for the development of Alzheimer’s disease.	329 (96.5)
28. It is safe for people with Alzheimer’s disease to drive, as long as they have a companion in the car at all times.	295 (86.5)
29. Alzheimer’s disease cannot be cured.	286 (83.9)
30. Most people with Alzheimer’s disease remember recent events better than things that happened in the past.	226 (66.3)

### GPs’ attitudes to dementia care

Table 7 shows the distribution of the GPs’ attitudes to dementia care. The overall mean score of dementia attitudes was 36.25 (SD = 5.12) out of 50 (72.5%). Most of the GPs agreed that “Families would rather be told about their relative’s dementia as soon as possible”, and “Dementia is best diagnosed by specialist services”, with the highest mean scores, while the mean scores of the items “There is little point in referring families to services as they do not want to use them” and “The primary care team has a very limited role to play in the care of people with dementia” were the lowest.

**Table 7:** GPs’ attitudes to dementia care (*n* = 341)

Items	Number of GPs strongly agree (%)	Number of GPs agree (%)	Neither (%)	Number of GPs disagree (%)	Number of GPs strongly disagree (%)	Mean ± SD
1. Much can be done to improve the quality of life of caregivers of people with dementia.	43 (12.6)	133 (39.0)	110 (32.3)	38 (11.1)	17 (5.0)	3.43 ± 1.01
2. Families would rather be told about their relative’s dementia as soon as possible.	124 (36.4)	173 (50.7)	23 (6.7)	12 (3.5)	9 (2.6)	4.15 ± 0.89
3. Much can be done to improve the quality of life of people with dementia.	47 (13.8)	127 (37.2)	110 (32.3)	41 (12.0)	16 (4.7)	3.43 ± 1.02
4. Providing diagnosis is usually more helpful than harmful.	106 (31.1)	170 (49.9)	44 (12.9)	12 (3.5)	9 (2.6)	4.03 ± 0.90
5. Dementia is best diagnosed by specialist services.	179 (52.5)	120 (35.2)	23 (6.7)	10 (2.9)	9 (2.6)	4.32 ± 0.92
6. Patients with dementia can be a drain on resources with little positive outcome.	7 (2.1)	30 (8.8)	64 (18.8)	165 (48.4)	75 (22.0)	3.79 ± 0.95
7. It is better to talk to the patient in euphemistic terms.	60 (17.6)	195 (57.2)	54 (15.8)	21 (6.2)	11 (3.2)	3.80 ± 0.91
8. Managing dementia is more often frustrating than rewarding.	17 (5.0)	54 (15.8)	85 (24.9)	128 (37.5)	57 (16.7)	3.45 ± 1.09
9. There is little point in referring families to services as they do not want to use them.	13 (3.8)	94 (27.6)	99 (29.0)	107 (31.4)	28 (8.2)	3.13 ± 1.03
10. The primary care team has a very limited role to play in the care of people with dementia.	31 (9.1)	126 (37.0)	102 (29.9)	69 (20.2)	13 (3.8)	2.73 ± 1.01

### GPs’ self-confidence on their dementia care skills

The details of GPs’ self-confidence on their dementia care skills were showed in Table 8. The overall mean score of GPs’ self-confidence on their dementia care skills was 53.93 (SD = 9.57) out of 75 (71.9%). Most GPs chose “I can do it probably” or “I can do it very well” on “Can be alert to the possibility of dementia in patients with cognitive impairment symptoms” and “Can refer the suspected dementia patients to a specialist if necessary”. Meanwhile these two items had the highest mean score of all the 15 items, while the scores of the item “Can provide non-drug guidance on improving cognitive function for dementia patients” and “Can provide guidance on behavioral symptoms for dementia patients” were the lowest.

**Table 8:** GPs’ self-confidence on their dementia care skills (*n* = 341)

Items	NCD (%)	Mean ± SD
Pre-diagnosis skills		3.63 ± 0.69
1. Can be alert to the possibility of dementia in patients with cognitive impairment symptoms.	255 (74.8)	3.86 ± 0.70
2. Can use MMSE (Mini-mental State Examination) for cognitive assessment.	202 (59.2)	3.58 ± 0.90
3. Can use CDT (Clock Drawing Test) for cognitive assessment.	183 (53.7)	3.49 ± 0.90
4. Can use IADL (Instrumental Activity of Daily living scale) for activity of daily living assessment.	200 (58.7)	3.57 ± 0.89
5. Can use NPI-Q (Neuropsychiatric Inventory Questionnaire) for behavioral and psychotic symptoms of dementia (BPSD) assessment.	157 (46.0)	3.34 ± 0.96
6. Can use GDS (Geriatric Depression Scale) for depression assessment.	184 (54.0)	3.50 ± 0.88
7. Can refer the suspected dementia patients to a specialist if necessary.	281 (82.4)	4.07 ± 0.72
Post-diagnosis skills		3.57 ± 0.73
8. Can do drug management for patients confirmed dementia.	191 (56.0)	3.48 ± 0.99
9. Can provide non-drug guidance on improving cognitive function for dementia patients, such as cognitive stimulation therapy, etc.	166 (48.7)	3.40 ± 0.94
10. Can provide guidance on behavioral symptoms for dementia patients, such as music therapy, etc.	173 (50.7)	3.43 ± 0.96
11. Can provide guidance on improving the ability of daily life for dementia patients, such as use of telephones, etc.	195 (57.2)	3.57 ± 0.85
12. Can provide guidance on safety for dementia patients, such as in-home safety, out-home safety, medication safety, etc.	224 (65.7)	3.73 ± 0.77
13. Can provide nursing guidance on nutritional support and complications prevention in patients with end-stage dementia.	219 (64.2)	3.71 ± 0.79
14. Can provide psychological guidance to caregivers.	196 (57.5)	3.60 ± 0.85
15. Can recommend resources of social support services available to caregivers, such as day care 2services, communication platform for family members of patients with Alzheimer’s disease, etc.	195 (57.2)	3.60 ± 0.85

NCD, the number of GPs choosing “I can do it probably” and “I can do it very well” was defined as “the number can do the job (NCD)”

### Association between the knowledge scores, attitudes scores and skills scores

A positive association remained between the knowledge scores and the attitude scores (*ρ* = 0.159, *P* = 0.003), and a similar association was reported between the scores of attitude and skills (*ρ* = 0.120, *P* = 0.027).

## Discussion

In the past 10 years, many countries have increasingly realized the seriousness of the dementia problem and the need for action. Some countries have developed strategies, policies, plans or guidelines for dementia to improve early diagnosis and long-term care. GPs should play an important role in improving early identification of dementia, especially in countries with large dementia population but insufficient specialists [31].

In England, 96% of patients were diagnosed or suspected with dementia in primary care settings, and two-thirds of them could be referred to specialists for further treatment quickly [32]. In Germany, the diagnosis rate of dementia in primary care was 40%. Despite a more favorable awareness towards dementia, the identification rate in primary care still needs to be improved [33, 34]. However, in lower-middle-income countries, family members of dementia patients rarely seek help from GPs and the GPs rarely encounter dementia cases [35]. In Beijing, the majority of the patients in urban CHSCs were the elderly [36–38], indicating that there might be more dementia patients. But most GPs in this study reported very few dementia cases they met. Patients and their families always failed to identify the typical dementia syndrome since they could not separate it from normal manifestation of aging. Among those who were able to identify the dementia patients, they would barely seek for help because of stigma. The perceived low quality of service in primary care might be a major reason why people prefer hospital care. The results of ADKS showed that GPs might misunderstand some syndrome of dementia, which meant that some GPs would fail to identify dementia patients. Therefore, it is necessary to strengthen the knowledge about dementia in the public, improve the recognition ability of GPs by professional training, and encourage patients to seek help from GPs in CHSCs.

Only 15.2% of the respondents had participated in relevant training of dementia in the past year in our study. And about 43.1% of participants (*n* = 147) agreed with an incorrect statement that “Once people have Alzheimer’s disease, they are no longer capable of making informed decisions about their own care”. This is evidence that GPs need to know about care giving in order to enable people with dementia to maintain autonomy and independence. The reason might be that the insufficient training opportunities provided by relevant organizations or the GPs themselves lack enthusiasm for learning. It was shown that even professional dementia care facilities, the training for staff to gain knowledge and abilities in dementia care were insufficient [39, 40]. They might not fulfil their roles in providing access to services in an appropriate way due to insufficient knowledge about dementia. There were positive relationships in both the knowledge and skills scores with the attitudes scores in our study, which might reveal that a positive attitude could encourage a GP to strength his knowledge and skills. Evidence proved that GPs could benefit from the professional dementia training [34]. Hence, the focus in the future is to improve the enthusiasm of GPs, and provide opportunities for them to receive professional training. These are important ways to improve positive attitudes for GPs toward managing dementia.

More than half of GPs agreed “Much can be done to improve the quality of life of dementia patients” and “Providing diagnosis is usually more helpful than harmful”. But their attitudes were more inclined to diagnose patients with dementia by specialists, and to deny the role of GPs in the management of dementia. The reason might be the lack of appropriate medical resources and a good connection with the specialist. It is necessary to perfect the resources of CHSCs, and improve the communication and referral system between CHSCs and hospitals.

More-general expectations were described that GPs have generally positive attitudes despite the low levels of dementia knowledge. The cause might be that most GPs recognized the significance of dementia diagnosis, management and caregiving [20, 29, 41]. Our study revealed that GPs in Beijing demonstrated more negative attitudes toward the role of primary care teams in dementia care in their responses to the DCAS statement “The primary care team has a limited role to play in the care of people with dementia” (item 10) compared to their counterparts in UK [29]. This finding might indicate that GPs in this study did not tend to regard dementia care as part of their professional duties. Moreover, our study revealed that more GPs agreed that managing dementia was more often frustrating compared to those in primary care in UK [41]. This is not surprising. Because the main tasks of CHSCs in China are providing care for people with hypertension, diabetes, psychosis and tuberculosis, not for dementia patients.

Our study showed that GPs had less confident in recognition of dementia. One of the reasons is likely to be the lack of knowledge of adequate screening tools. GPs especially had significant difficulties in the use of NPI-Q, which is one of the most common professional scales used in BPSD assessment. Over 50% of GPs were not confident on this item. The potential reason might be the screening tools are not widely used in community. Identifying dementia by screening tools in primary care could improve diagnosis of dementia and minimize the poor outcomes due to late intervention [42, 43]. The professional training on screening tools for dementia could help to improve GPs’ ability of identifying dementia patients.

The score of post-diagnosis skills was lower than that of pre-diagnosis skills, which was similar to the results from Turner’ s study [29]. It indicated that GPs had lower confidence in patients’ post-diagnosis management, compared with their confidence in identification of dementia. The home-based patients and their caregivers needed psychosocial support, nursing services and professional care guidance on addressing patient’s BPSD, improving patient’s cognitive function and maintaining the patient’s independence in daily life [19, 44]. But the GPs in China have limited knowledge and skills about dementia care due to the shortage of education programs and support services. To meet these needs of the patients and caregivers, detailed dementia care handbook, adequate dementia knowledge training and continuing professional development programs are needed for GPs. Dementia management should be covered in the national basic package of public health services, so that GPs might be encouraged.

Moreover, nursing treatment, psychosocial counselling and social support are major needs of dementia patients and caregivers [44]. Many developed countries and regions have established supportive policies for dementia patients and integrated management system consisted of community care, home nursing and health treatment [23]. An integrated care system including hospitals, CHSCs and social care organizations should be developed in China. We believe that the integration of medical and social services could alleviate the physical and mental burden of caregivers [45].

Important aspects regarding dementia care of GPs were revealed in this study. The results and conclusions in this study could serve as a basis for future community-based care. This study also has some limitations. First, a cross-sectional design of the present study did not allow the determination of causal relationships, only associations between knowledge and attitudes. Second, the staff population in the 6 districts may not reflect CHSCs in other rural parts of Beijing or other parts of China when generalizing the findings.

## Conclusion

GPs demonstrate low levels of dementia knowledge and skills, but express generally positive attitudes towards dementia in this study. Therefore, detailed dementia care handbook, adequate dementia knowledge training or continuing professional development programs for GPs, and to translate them into practice to improve care outcomes for people with dementia is much needed in China. In addition, dementia management should be covered in the national basic package of public health services in primary care.

## Data Availability

The datasets generated and analyzed during the current study are not publicly available to protect participant privacy, but are available from the corresponding author on reasonable request.

## Abbreviations

GPs: General practitioners
CHSCs: Community Health Service Centers
ADKS: Alzheimer’s Disease Knowledge Scale
DCAS: Dementia Care Attitude Scale
SD: Standard deviations
WHO: World Health Organization
IQR: Inter-quartile range
IBM-SPSS: IBM Statistical Package for Social Science software program
NCD: The number of GPs choosing “I can do it probably” and “I can do it very well” was defined as “the number can do the job (NCD)”
BPSD: Behavioral and psychotic symptoms of dementia

## References

[1] Evans-Lacko S, Bhatt J, Comas-Herrera A, D’Amico F, Farina N, Gaber S, et al. World Alzheimer report 2019: new Frontiers. In: The global voice on dementia: Alzheimer’s disease international; 2019. https://www.alz.co.uk/research/WorldAlzheimerReport2019.pdf. Accessed 3 May 2020.

[2] Huang Y, Wang Y, Wang H, Liu Z, Yu X, Yan J (2019). Prevalence of mental disorders in China: a cross-sectional epidemiological study. Lancet Psychiatry.

[3] Wang YQ, Jia RX, Liang JH, Li J, Qian S, Li JY (2019). Dementia in China (2015-2050) estimated using the 1% population sampling survey in 2015. Geriatr Gerontol Int.

[4] Dreier-Wolfgramm A, Michalowsky B, Austrom MG, van der Marck MA, Iliffe S, Alder C (2017). Dementia care management in primary care: current collaborative care models and the case for interprofessional education. Z Gerontol Geriatr.

[5] Thyrian JR, Hertel J, Wucherer D, Eichler T, Michalowsky B, Dreier-Wolfgramm A (2017). Effectiveness and safety of dementia care management in primary care: a randomized clinical trial. JAMA Psychiatry.

[6] Reilly S, Miranda-Castillo C, Malouf R, Hoe J, Toot S, Challis D (2015). Case management approaches to home support for people with dementia. Cochrane Database Syst Rev.

[7] Galvin JE, Sadowsky CH (2016). Practical guidelines for the recognition and diagnosis of dementia. J Am Board Fam Med.

[8] British Columbia Ministry of Health (2016). Cognitive impairment: recognition, diagnosis and management in primary care.

[9] California Workgroup on Guidelines for Alzheimer’s Disease Management (2008). Guideline for Alzheimer’s disease management.

[10] Thyrian JR, Hoffmann W (2012). Dementia care and general physicians-a survey on prevalence, means, attitudes and recommendations. Cent Eur J Public Health.

[11] Subramaniam M, Ong HL, Abdin E, Chua BY, Shafie S, Siva Kumar FD (2018). General practitioner’s attitudes and confidence in managing patients with dementia in Singapore. Ann Acad Med Singap.

[12] Beccaro M, Lora Aprile P, Scaccabarozzi G, Cancian M, Costantini M (2013). Survey of Italian general practitioners: knowledge, opinions, and activities of palliative care. J Pain Symptom Manag.

[13] Ahmad S, Orrell M, Iliffe S, Gracie A (2010). GPs’ attitudes, awareness, and practice regarding early diagnosis of dementia. Br J Gen Pract.

[14] Veneziani F, Panza F, Solfrizzi V, Capozzo R, Barulli MR, Leo A (2016). Examination of level of knowledge in Italian general practitioners attending an education session on diagnosis and management of the early stage of Alzheimer’s disease: pass or fail?. Int Psychogeriatr.

[15] Foley T, Boyle S, Jennings A, Smithson WH (2017). “We’re certainly not in our comfort zone”: a qualitative study of GPs’ dementia-care educational needs. BMC Fam Pract.

[16] Wang J, Xiao LD, Li X (2019). Health professionals’ perceptions of developing dementia services in primary care settings in China: a qualitative study. Aging Ment Health.

[17] National Health Commission of the People’s Republic of China (2017). National basic public health service regulations (the third edition).

[18] Chen Z, Yang X, Song Y, Song B, Zhang Y, Liu J (2017). Challenges of dementia Care in China. Geriatrics (Basel).

[19] Wang M, Shao S, Li J, Liu Y, Xu X, Du J (2018). The needs of informal caregivers and barriers of primary care workers toward dementia management in primary care: a qualitative study in Beijing. BMC Fam Pract.

[20] Wang Y, Xiao LD, Luo Y, Xiao SY, Whitehead C, Davies O (2018). Community health professionals' dementia knowledge, attitudes and care approach: a cross-sectional survey in Changsha, China. BMC Geriatr.

[21] Xu X, Liu B (2017). Review on the research of social support on old dementia patient. Chin J Soc Med.

[22] World Health Organization (2012). Dementia. A public health Priority.

[23] Patterson M, Comas-Herrera A, Knapp M, Guerchet M, Karagiannidou M. World Alzheimer Report 2016: Improving Healthcare. In: The global voice on dementia: Alzheimer’s disease international; 2016. https://www.alz.co.uk/research/ WorldAlzheimerReport2016. Accessed 3 May 2020.

[24] The Research Group of Beijing on Aging (2018). White paper on Beijing’s aged career development and pension system construction.

[25] National Health Commission of the People’s Republic of China (2015). Beijing: There are 150 thousand to 200 thousand dementia patients, many hospitals have memory loss clinics.

[26] Central People’s Government of the People’s Republic of China (2017). There are 3.29 million elderly people registered in Beijing, and the aging population has exceeded 24%.

[27] Carpenter BD, Balsis S, Otilingam PG, Hanson PK, Gatz M (2009). The Alzheimer’s disease knowledge scale: development and psychometric properties. Gerontologist..

[28] He RL, Jing CL, Li BE, Pang GF, Yu HM, Sun L (2013). Reliability and validity of the Chinese version of the Alzheimer’s disease knowledge scale. Chin J Nurs.

[29] Turner S, Iliffe S, Downs M, Wilcock J, Bryans M, Levin E (2004). General practitioners’ knowledge, confidence and attitudes in the diagnosis and management of dementia. Age Ageing.

[30] Yu L, Xu Q, Wang ZY, Cao WW, Mi JH, Li YS (2013). A survey on the attitudes and confidence of general practitioners in handling dementia and its related problems. Natl Med J China.

[31] World Health Organization (2013). Comprehensive mental health action plan 2013-2020.

[32] Wilcock J, Iliffe S, Turner S, Bryans M, O’Carroll R, Keady J (2009). Concordance with clinical practice guidelines for dementia in general practice. Aging Ment Health.

[33] Eichler T, Thyrian JR, Hertel J, Köhler L, Wucherer D, Dreier A (2014). Rates of formal diagnosis in people screened positive for dementia in primary care: results of the DelpHi-trial. J Alzheimers Dis.

[34] Thyrian JR (2017). People with dementia in primary care: prevalence, incidence, risk factors and interventions. Z Gerontol Geriatr.

[35] Prince M, Livingston G, Katona C (2007). Mental health care for the elderly in low-income countries: a health systems approach. World Psychiatry.

[36] Yu H, Wei Y, Jin G, Zhao Y, Lu X (2019). Survey on consultation length and waiting time of community general practice clinics. Chin J Gen Pract.

[37] Feng S, Li W, Lu X (2013). Influencing factors of patients’ satisfaction with community health Services in Xicheng District of Beijing. Chin Gen Prac.

[38] Yuan Q, Yan H, Zhu Q, Wang Q, Liang C, Chang Y (2009). Patient satisfaction on community health Services in Beijing - qualify of care as patient perspective. Chin Gen Prac.

[39] Gould E, Cox T, Johnson MA, Vaillancourt EM, Stanley A (2010). Dementia care training in nursing homes and assisted living settings perspectives from the Alzheimer’s association based on the evaluation of foundations of dementia care. Alzheimer’s Care Today.

[40] Bamford C, Poole M, Brittain K, Chew-Graham C, Fox C, Iliffe S (2014). Understanding the challenges to implementing case management for people with dementia in primary care in England: a qualitative study using normalization process theory. BMC Health Serv Res.

[41] Bryans M, Keady J, Turner S, Wilcock J, Downs M, Iliffe S (2003). An exploratory survey into primary care nurses and dementia care. Br J Nurs.

[42] Yokomizo JE, Simon SS, Bottino CM (2014). Cognitive screening for dementia in primary care: a systematic review. Int Psychogeriatr.

[43] Eichler T, Thyrian JR, Hertel J, Michalowsky B, Wucherer D, Dreier A (2015). Rates of formal diagnosis of dementia in primary care: the effect of screening. Alzheimers Dement (Amst).

[44] Eichler T, Thyrian JR, Hertel J, Richter S, Wucherer D, Michalowsky B (2016). Unmet needs of community-dwelling primary care patients with dementia in Germany: prevalence and correlates. J Alzheimers Dis.

[45] Jia L, Quan M, Fu Y, Zhao T, Li Y, Wei C (2020). Dementia in China: epidemiology, clinical management, and research advances. Lancet Neurol.

